# Targeted high volume hemofiltration could avoid extracorporeal membrane oxygenation in some patients with severe Hantavirus cardiopulmonary syndrome

**DOI:** 10.1002/jmv.26930

**Published:** 2021-03-23

**Authors:** René López, Rodrigo Pérez‐Araos, Álvaro Salazar, Mauricio Espinoza, Cecilia Vial, Analia Cuiza, Pablo A. Vial, Jerónimo Graf

**Affiliations:** ^1^ Departamento de Paciente Crítico Clínica Alemana de Santiago Santiago Chile; ^2^ Carrera de Medicina, Facultad de Medicina Clínica Alemana Universidad del Desarrollo Santiago Chile; ^3^ Carrera de Kinesiología, Facultad de Medicina Clínica Alemana Universidad del Desarrollo Santiago Chile; ^4^ Programa Hantavirus, Instituto de Ciencias e Innovación en Medicina (ICIM), Facultad de Medicina Clínica Alemana Universidad del Desarrollo Santiago Chile; ^5^ Departamento de Pediatría Clínica Alemana de Santiago Santiago Chile

**Keywords:** Andes Hantavirus, hantavirus cardiopulmonary syndrome, hantavirus pulmonary syndrome, high volume hemofiltration, transpulmonary thermodilution, venoarterial extracorporeal membrane oxygenation

## Abstract

**Background:**

Hantavirus cardiopulmonary syndrome (HCPS) has a high lethality. Severe cases may be rescued by venoarterial extracorporeal membrane oxygenation (VA ECMO), alongside substantial complications. High volume hemofiltration (HVHF) is a depurative technique that provides homeostatic balance allowing hemodynamic stabilization in some critically ill patients.

**Methods:**

We implemented HVHF before VA ECMO consideration in the last five severe HCPS patients requiring mechanical ventilation and vasoactive drugs admitted to our intensive care unit. Patients were considered HVHF‐responders if VA ECMO was avoided and HVHF‐nonresponders if VA ECMO support was needed despite HVHF. A targeted‐HVHF strategy compounded by aggressive hyperoncotic albumin, sodium bicarbonate, and calcium supplementation plus ultrafiltration to avoid fluid overload was implemented on three patients.

**Results:**

Patients had maximum serum lactate of 8.8 (8.7–12.8) mmol/L and a lowest cardiac index of 1.8 (1.8–1.9) L/min/m^2^. The first two required VA ECMO. They were connected later to HVHF, displayed progressive tachycardia and declining stroke volume. The opposite was true for HVHF‐responders who received targeted‐HVHF. All patients survived, but one of the VA ECMO patients suffered a vascular complication.

**Conclusion:**

HVHF may contribute to support severe HCPS patients avoiding the need for VA ECMO in some. Early connection and targeted‐HVHF may increase the chance of success.

**Abbreviations**
ANDV: Andes Hantavirus
CI: cardiac index
ECF: extracellular fluid
ECMO: extracorporeal membrane oxygenation
ELISA: enzyme‐linked immunosorbent assay
EVLWI: extravascular lung water index
FO: fluid overload
HCPS: hantavirus cardiopulmonary syndrome
HVHF: high volume hemofiltration
ICU: intensive care unit
IMV: invasive mechanical ventilation
ITBVI: intrathoracic blood volume index
PEEP: positive end‐expiratory pressure
PVP: pulmonary vascular permeability
PVPI: pulmonary vascular permeability index
RT‐qPCR: specific reverse‐transcription polymerase chain reaction
SI: stroke index
SOFA: sequential organ failure assessment
SVV: stroke volume variation
TPTD: transpulmonary thermodilution
UF: ultrafiltration
VA ECMO: venoarterial extracorporeal membrane oxygenation

## 1 INTRODUCTION

Andes Hantavirus (ANDV) is an orthohantavirus, member of the Hantaviridae family, endemic in Chile and Argentina, and its main reservoir is the long‐tailed pygmy rice rat (*Oligoryzomys longicaudatus*).^1^ Humans are infected primarily by the inhalation of aerosolized excreta from infected rodents.^2^ Additionally, ANDV is the only Hantavirus known to be transmissible between humans.^3^ The incubation period of ANDV varies from 7 to 39 days^4^ followed by a cardiopulmonary phase that evolves from dry cough to respiratory failure due to capillary leak into the pulmonary interstitium. Noncardiogenic pulmonary edema is evidenced by chest radiographs showing peribronchial haze and Kerley's B lines that subsequently progress to alveolar flooding with proteinaceous fluid.^5^ Hantavirus cardiopulmonary syndrome (HCPS) also includes circulatory shock compounded by hypovolemia and myocardial depression.^6^ Using transpulmonary thermodilution (TPTD), we recently documented that increased pulmonary vascular permeability (PVP) was associated to hypovolemia and systolic dysfunction in HCPS patients.^7^ Patients with severe HCPS may ultimately develop refractory hypoxemia and/or circulatory shock that leads to death in up to 35–40% of patients, making HCPS is one of the deadliest infectious diseases.^8^, ^9^, ^10^, ^11^ Unfortunately there are no drugs with proven efficacy for HCPS and treatment is based on critical care support including judicious fluid management, vasoactive drugs, invasive mechanical ventilation (IMV), and extracorporeal support with venoarterial extracorporeal membrane oxygenation (VA ECMO) in refractory cases.^9^, ^12^, ^13^, ^14^

High volume hemofiltration (HVHF) is a form of depurative therapy that has been used as adjunctive support for refractory septic shock.^15^, ^16^, ^17^ As opposed to hemodialysis where depuration is attained by diffusion of small molecules, in hemofiltration, blood is cleared of small and medium‐size molecules by convection.^18^ The intensity of depuration is set by the filtration or replacement rate; conventional renal replacement rates are 25–35 ml/kg/h, whereas HVHF uses 50–100 ml/kg/h.^19^ The exact mechanism of hemodynamic improvement with HVHF in septic shock is unclear and three main mechanisms have been suggested (1) removal of ill‐defined vasoactive or myocardial depressant factors^20^; (2) immune modulation through plasmatic cytokine peak amputation or their mobilization from tissues^19^, ^21^; and (3) internal homeostasis restoration (acid–base, temperature) devoid of fluid and sodium overload.^22^ Despite safety and hemodynamic benefit frequently seen with HVHF in septic shock models^20^, ^23^ and clinical case series,^15^, ^16^, ^17^, ^19^ randomized clinical trials have not shown consistent improvements in outcome^24^; thus HVHF remains a rescue therapy for refractory septic shock. There are only two reports on the use of depurative therapies in Hantavirus pulmonary syndrome. In 2006, Seitsonen et al.^25^ reported the successful use of continuous venovenous hemodiafiltration in two patients with Puumala virus pulmonary syndrome. Ten years later, Bugedo et al.^26^ reported a patient with ANDV severe HCPS successfully supported with HVHF.

We present the first clinical case series of patients with ANDV severe HCPS supported with HVHF in addition to vasoactive drugs and IMV, before VA ECMO consideration. Success or failure of HVHF was stratified according to the subsequent need for VA ECMO support.

## 2 METHODS

### 2.1 Study design and patients

This is an observational retrospective case series report; patients were not treated according to a research protocol. The cohort is part of a prospectively obtained database by the Hantavirus program from the *Instituto de Ciencias e Innovación en Medicina, Facultad de Medicina Clínica Alemana‐Universidad del Desarrollo*. Our local IRB and ethics committee approved this registry (ID19, September 29th 2011).

For this study, only patients with HCPS admitted to our adult intensive care unit (ICU) and supported with IMV and HVHF were included. The diagnosis of HCPS was confirmed by ANDV specific reverse‐transcription polymerase chain reaction (RT‐qPCR) using an in‐house PCR as described in Vial et al.^27^ With this technique we quantified viral genome and determined viremia from the buffycoat. In three patients we also performed quantitative enzyme‐linked immunosorbent assay (ELISA) detecting ANDV specific immunoglobulin M. Demographic, clinical, laboratory, hemodynamic, and pulmonary monitoring data, as well as hemofiltration and VA ECMO support variables and relevant outcomes, were collected using a standardized case record form. Anonymized data was then entered into a dedicated database. Data are presented as median and interquartile range (IQR), where appropriate.

### 2.2 Variables of interest

Patients were stratified as HVHF‐responders if VA ECMO was avoided and HVHF‐nonresponders if VA ECMO support was needed or death ensued. Hemodynamic, respiratory and laboratory data including variables from TPTD monitoring, oxygenation, blood lactate, vasoactive drug, and reanimation fluids requirements were recorded in reference to HVHF onset. Time course of hemodynamic and respiratory variables aligned to HVHF onset and grouped according to HVHF responsiveness is presented. Specific data on HVHF, such as hemofilter type, circuit blood flow, replacement fluid rate, and net ultrafiltration (UF) volume were recorded.

Transpulmonary thermodilution was performed with the PiCCO system (PULSION Medical Systems AG, Munich, Germany). This monitoring technique allows to measure cardiac index (CI), stroke index (SI), volumetric cardiac preload (intrathoracic blood volume index [ITBVI]), preload dependency of stroke volume (stroke volume variation [SVV]), pulmonary edema (extravascular lung water index [EVLWI]) and pulmonary permeability (pulmonary vascular permeability index, [PVPI]). In HVHF‐nonresponders, these variables were available only initially, as TPTD is invalid once on VA ECMO due to tracer removal. A detailed explanation of the TPTD technique in this setting can be found in our previous report on the topic.^7^

### 2.3 Hemofiltration procedure

All hemofiltration procedures were performed with a Diapact continuous renal replacement therapy machine (B. Braun Avitum AG, Melsungen, Germany) using a Diacap Acute L hemofilter (B. Braun). These are polysulfone hollow‐fiber membranes with a surface area of 2 m^2^, a sieving coefficient of 0.55 for myoglobin (molecular weight 17,000 Dalton), and an ultrafiltration coefficient of 58 ml/h/mmHg. Replacement fluid was administered before the hemofilter (predilution) using Priosol (B. Braun), a bicarbonate‐based solution with the following composition: sodium 140 mmol/L, calcium 1.5 mmol/L, magnesium 0.5 mmol/L, chloride 109 mmol/L, bicarbonate 35 mmol/L, glucose 1 g/L, and osmolarity 292 mOsm/L. Potassium was added to maintain the plasma concentration between 4 and 5 mmol/L. All received HVHF with replacement flows (Qr) greater than 50 ml/kg/h for at least 6 h. Circuit blood flows (Qb) between 200 and 270 ml/min were used, without anticoagulation, through a 14 Fr, 20 cm long double lumen hemodialysis catheter (Duo‐Flow 400XL, Medcomp) placed in a femoral vein.

## 3 RESULTS

**Figure 1:**
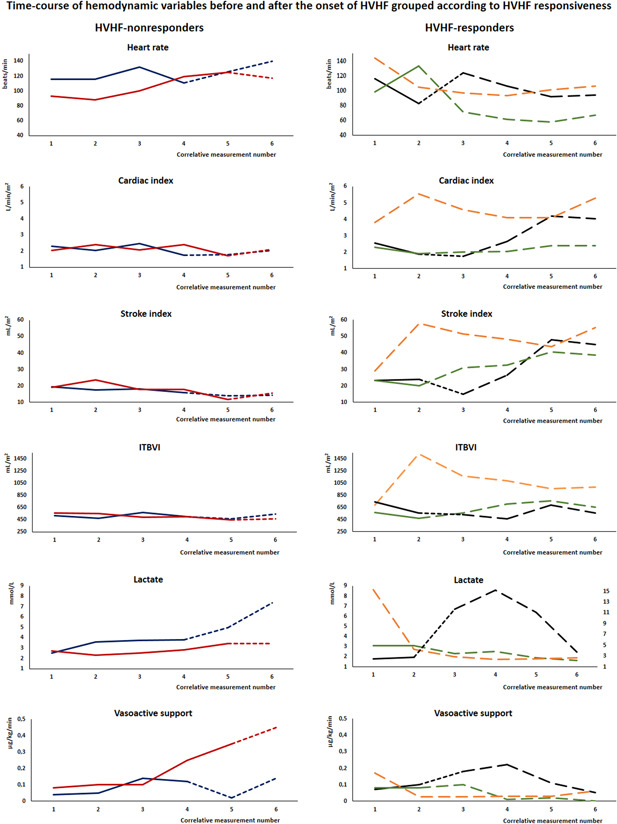
HVHF‐nonresponders required VA ECMO. Each color represents a single patient: patient 1, blue; patient 2, red; patient 3, black; patient 4, green; patient 5, orange. The period before the onset of HVHF is marked with continuous lines. The period of HVHF is marked with dashed lines; standard HVHF is marked with short‐dashed lines and targeted HVHF marked with long‐dashed lines. Patient 3 has both of them, and patient 5 has only a targeted HVHF period. The abscissa is time and the numbers on it indicate correlative measurements rather than strict units of time. The doses of all vasoactive drugs used (noradrenaline, adrenaline, dobutamine, and milrinone in μg/kg/min) at the moment of each transpulmonary thermodilution measurement were added to summarize vasoactive support at each time point. HVHF, high volume hemofiltration; ITBVI, intrathoracic blood volume index; VA ECMO, venoarterial extracorporeal membrane oxygenation

**Figure 2:**
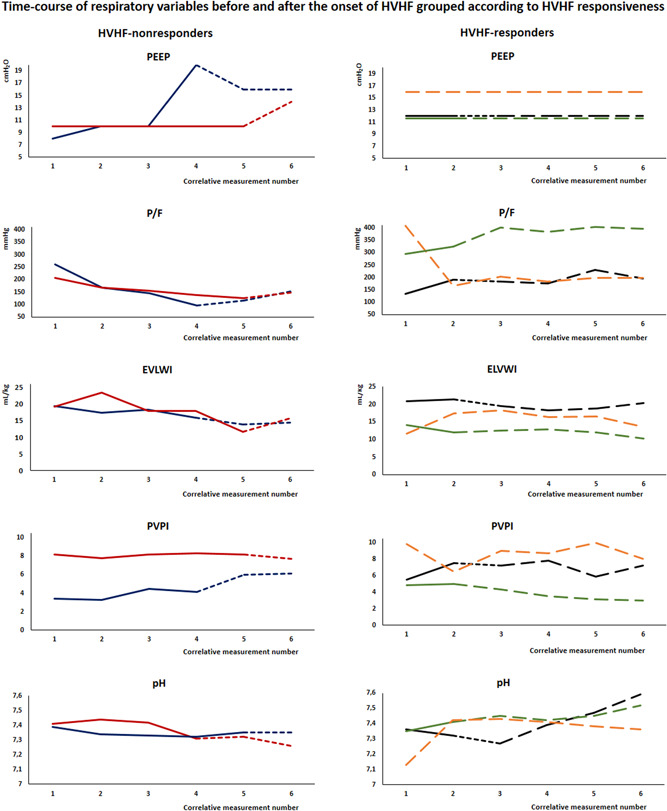
HVHF‐nonresponders required VA ECMO. Each color represents a single patient: patient 1, blue; patient 2, red; patient 3, black; patient 4, green; patient 5, orange. The period before the onset of HVHF is marked with continuous lines. The period of HVHF is marked with dashed lines; standard HVHF is marked with short‐dashed lines and targeted HVHF marked with long‐dashed lines. Patient 3 has both of them, and patient 5 has only a targeted HVHF period. The abscissa is time and the numbers on it indicate correlative measurements rather than strict units of time. EVLWI, extravascular lung water index; HVHF, high volume hemofiltration; PEEP, positive end‐expiratory pressure; P/F, arterial oxygen partial pressure to fraction of inspired oxygen ratio; PVPI, permeability vascular pulmonary index; VA ECMO, venoarterial extracorporeal membrane oxygenation

We identified five patients with severe HCPS supported with HVHF before considering VA ECMO support between February and December 2017. These patients are part of the cohort of 11 patients recently reported to describe the TPTD pattern of HCPS.^7^ They were all young previously healthy patients, four were male. Individual demographic, virological, laboratory, severity scoring, hemodynamic, and respiratory data are shown in Tables 1 and 2. Individual time course of hemodynamic and respiratory variables aligned to HVHF onset and grouped according to HVHF responsiveness are shown in Figures 1 and 2. All of them had a circulatory shock with vasoactive drug requirement and lactic acidosis (Tables 1 and 2). They all received hydrocortisone at stress doses with an initial bolus of 100 mg, followed by 50 mg every 6 h. All of them also had an acute respiratory failure with IMV and moderate‐to‐high positive end‐expiratory pressure (PEEP) requirement (Table 2) under deep sedation with continuous infusions of midazolam and fentanyl as well as neuromuscular blockade with cisatracurium. All of them had a severe multiorgan failure, with the highest sequential organ failure assessment (SOFA) score of 12^10^, ^11^, ^12^, ^13^ points (Table 1). All patients received an intravenous infusion of convalescent immune plasma at an ANDV neutralizing antibody dose of 5000 U/kg within 15 h of admission according to our local protocol.^10^ We did not use normal intravenous immunoglobulin. Individual HVHF settings together with the amount of hyperoncotic albumin, sodium bicarbonate and calcium intravenously added within the first hours of HVHF are shown in Table 3. Individual changes in laboratory and TPTD variables induced after 6 to 15 h of HVHF are shown in Table S1. Patients are presented chronologically.

**Table 1:** Demographic, virological, severity scoring, laboratory, and clinical data for each patient

	HVHF‐nonresponders			HVHF‐responders		
	1	2	3	4	5^a^	Median (IQR)
Age, years	15	15	17	22	29	17 (15–22)	
Weight, kg	64	63	67	72	81	67 (64–72)	
IgM ANDV	n/a	+	+	n/a	+	–	
Viremia, copies/ml blood	2.4 × 10^5^	2.7 × 10^4^	8.2 × 10^3^	4.1 × 10^4^	undetectable	–	
Prodromal period, days	5	4	5	7	6	5 (5,6)	
APACHE II score, points	10	17	14	8	14	14 (10–14)	
Admission SOFA score, points	4	11	10	9	11	10 (9–11)	
Lowest platelet count, 10^3^/mm^3^	37	27	42	49	85	42 (37–49)	
Highest hematocrit, %	48.9	48.4	55.8	44.3	47	48.4 (47‐48.9)	
Highest serum lactate, mmol/L	12.8	8.8	15.2	3.1	8.7	8.8 (8.7‐12.8)	
Highest SOFA score, points	12	14	10	10	12	12 (10–12)	
Admission to intubation interval, h	17	2	1	1	−24^b^	–	
Admission to immune plasma interval, h	15	2	4	1.5	1	2 (1.5‐4)	
Time on IMV, days	9	10	4	2	4	4 (4–9)	
ICU‐LOS, days	14	18	6	3	4	6 (4–14)	
Hospital‐LOS, days	20	87	13	6	4^c^	13 (6–20)	

Abbreviations: APACHE II, acute physiology and chronic health evaluation II; HVHF, high volume hemofiltration; ICU, intensive care unit; IgM ANDV, Andes Hantavirus specific immunoglobulin M; IMV, invasive mechanical ventilation; LOS, length of stay; n/a, nonavailable; SOFA, sequential organ failure assessment; +, positive test.

^a^ Patient 5 was transferred from and back to another hospital.

^b^ The negative time interval refers to intubation being before admission to our center.

^c^ Hospital‐LOS is the shortest because once extubated, this patient was transferred back to the referring center.

**Table 2:** Hemodynamic variables, transpulmonary thermodilution, vasoactive support, and respiratory variables for each patient

	HVHF‐nonresponders			HVHF‐responders		
Characteristic	1	2	3	4	5	Median [IQR]
Vital sings						
Highest HR, beats/min	148	138	124	111	145	138 (124–144)
Lowest MAP, mmHg	73	65	79	77	69	73 (69–77)
Highest RR, breaths/min	33	32	24	35	43	33 (32–35)
Transpulmonary thermodilution variables						
Lowest stroke index, ml/m^2^	13.9	11.6	15	20.1	29	15 (13.9–20.1)
Lowest cardiac index, L/min/m^2^	1.75	1.7	1.76	1.9	2.76	1.8 (1.8–1.9)
Lowest ITBVI, ml/m^2^	451	447	461	467	644	461 (451–467)
Highest EVLWI, ml/kg	15.9	25.3	21.3	14	18.2	18.2 (15.9–21.3)
Highest PVPI, dimensionless	6.4	8.3	7.8	5	10	7.8 (6.4–8.3)
Vasoactive support						
Adrenaline highest dose, μg/kg/min	0	0.03	0.18	0	0.2	0.03 (0–0.18)
Noradrenaline highest dose, μg/kg/min	0.16	0.45	0.18	0.15	0.04	0.16 (0.15–0.18)
Dobutamine highest dose, μg/kg/min	1	0	1	0	0	0 (0–1)
Milrinone highest dose, μg/kg/min	0.25	0	0.5	0	0	0 (0–0.25)
Respiratory variables						
LIS, points	3.75	2.75	2.75	2.5	2.25	2.8 (2.5–2.8]
Lowest P/F ratio, mmHg	97	125	133	295	166	133 (125–166]

Abbreviations: EVLWI, extravascular lung water index; HVHF, high volume hemofiltration; HR, heart rate; MAP, mean arterial pressure; IQR, interquartile range; ITBVI, intrathoracic blood volume index; LIS, lung injury score (1–4 points); PVPI, pulmonary vascular permeability index; RR, respiratory rate. LIS greater than 2.5 points has been considered diagnostic for the acute respiratory distress syndrome; P/F ratio, arterial oxygen partial pressure to fraction of inspired oxygen ratio.

**Table 3:** Hemofiltration variables and external addition of intravenous hyperoncotic albumin, bicarbonate, and calcium during HVHF

	HVHF‐nonresponders		HVHF‐responders			
Characteristic	1	2	3S	3T	4	5^a^
Intubation to HVHF onset interval, hours	30	15	11	20	8	27/3^b^
HVHF run, h	14	15	9	6	9	9
Qb, ml/min	230	200	200	200	270	250
Qr, ml/kg/h	78	71	60	60	56	74
Net ultrafiltered volume, ml	0	0	0	2,490	3,690	4,800
20% albumin addition, ml	50	200	0	400	200	400
Bicarbonate addition, mmol	266	67	500	583	166	500
Calcium addition, mmol	3.25	0	0	3.25	3.25	3.25

Abbreviations: HVHF, high volume hemofiltration; Qb, circuit blood flow; Qr, replacement flow; 3S, patient 3 during standard HVHF; 3T, patient 3 during targeted HVHF.

^a^ Patient 5 was transferred from another hospital.

^b^ This patient was intubated 24 h before admission to our center; HVHF started within 3 h of admission to our center.

Despite HVHF, the first two patients continued to deteriorate, developing circulatory failure as attested by declining SI, CI, ITBVI, and rising blood lactate levels while oxygenation worsened and they ultimately required VA ECMO support (Figures 1 and 2, Table S1). The second patient had a vascular complication during the femoral arterial cannulation for VA ECMO. This resulted in a large superinfected hematoma of the groin that required vascular and reconstructive surgery with a lengthy hospital stay (Table 1).

The third patient exhibited a dual behavior after HVHF initiation; initially, SI continued to decrease and CI was maintained at the expense of progressive tachycardia and increasing catecholamine doses, and lactate continued to rise (Figure 1, Table S1). After 9 h of ineffective standard HVHF, a trial of targeted HVHF was started. This modified approach consisted of aggressive fluid resuscitation with hyperoncotic albumin (20%, 400 ml) and sodium bicarbonate (1.4%, 1500 ml and 5.6%, 500 ml) in addition to calcium supplementation while aggressive UF was added to HVHF (Table 3). The target was to rapidly correct hypoalbuminemia and metabolic acidosis while keeping ionic calcemia in the upper normal range and avoiding fluid overload (FO) (Table 3 and Table S1). Shortly after this strategy was started, SI increased more than three times, CI rose more than two times and a prominent lactate washout curve followed (Figure 1, Table S1). Oxygenation impairment, lung edema, and increased pulmonary vascular permeability remained stable through the course of this dual hemodynamic course (Figure 2).

The fourth patient also received supplemental hyperoncotic albumin, sodium bicarbonate, and calcium while aggressive UF was added to HVHF (Table 3). He had a rapid hemodynamic improvement increasing SI with relatively small increases in ITBVI, reducing tachycardia and lactate levels (Figure 1 and Table S1). This patient had the least pulmonary involvement of this series. He improved oxygenation and slightly decreased EVLWI and PVPI throughout the course of HVHF (Figure 2).

The fifth patient was intubated 24 h before referral to our center and was transferred as a VA ECMO candidate. Upon arrival at our center, he received large intravenous fluid boluses of hyperoncotic albumin (20%, 400 ml), immune plasma (500 ml), and hypertonic sodium bicarbonate (5.6%, 750 ml) together with calcium supplementation while HVHF was started with a net UF rate of 800 ml/h (Table 3). Stroke index, CI, and ITBVI rapidly increased and then stabilized while lactate cleared (Figure 1, Table S1) at the cost of a transient increase in EVLWI and oxygenation impairment (Figure 2). These last three patients did not require VA ECMO support and were free of vascular complications. The last two had a fast uneventful recovery with only four days of IMV and less than a week of ICU stay. All the patients were discharged home, returned to their previous activities, and are still alive. The only sequela was intermittent claudication of one leg in the second patient.

## 4 DISCUSSION

To our best knowledge, this is the largest report on depurative therapy in severe HCPS. The main observation was the ability to successfully support severe circulatory and respiratory failure, avoiding VA ECMO connection in three of five severe HCPS patients in whom preemptive HVHF was used. Four of them had a CI < 2.0 L/min/m^2^ and four had a blood lactate level >4 mmol/L; both features of the VA ECMO connection criteria for severe HCPS established by Crowley et al.^12^ Responsiveness to HVHF in septic shock has been assessed by catecholamine requirement, cardiac output, and lactate clearance.^15^, ^16^, ^17^ We used VA ECMO need as comprehensive criteria of HVHF responsiveness in these patients.

The key question is why some patients improved hemodynamics while others continued to deteriorate, requiring VA ECMO to avoid demise. Nonresponders could be considered more severely ill on the basis of the greatest viremia (patient 1), lower platelet counts (Table 1), higher temperature (Table S1), lower SI (Table 2 and Table S1, Figure 1), the greatest severity of respiratory failure (patient 1; Table 2 and Figure 2) and the greatest pulmonary edema in the cohort (patient 2; Table 2 and Figure 2). Yet, severity scores, peak PVPI, hemoconcentration, and lactate levels were similar between HVHF‐responders and nonresponders. On the other hand, HVHF was given differently to responders and nonresponders; besides an earlier onset (Table 3), responders received targeted HVHF compounded by aggressive resuscitation with hyperoncotic albumin and sodium bicarbonate while calcium was supplemented and UF was added with the aim of normalizing plasma albumin, bicarbonate and ionic calcium while avoiding FO (Table 3 and Table S1). Patient 3 provides a good comparison of the standard and targeted HVHF approaches. Although connected early to standard HVHF, this patient initially seemed to follow the path of hemodynamic deterioration of patients 1 and 2, but when switched to targeted HVHF, SI rose, heart rate dropped and lactate washed out (Figure 1). We saw the same behavior on patients 4 and 5. The whole series seems to suggest a learning curve in terms of both progressively earlier connection and active HVHF optimization to swiftly restore homeostasis.

The first report of hemofiltration in a septic model showed reversal of myocardial depression ascribed to the removal of a filterable cardiodepressant factor.^20^ Then HVHF showed improved myocardial performance in an endotoxin‐induced shock model.^23^ Myocardial depression was reversed only by early hemofiltration in a pneumonia model, suggesting that timing could be important.^28^ In a case series of refractory hypodynamic septic shock subjected to HVHF nearly half reversed myocardial depression and survived.^15^ Similar to our HCPS series, earlier onset of HVHF was associated with a positive response.^15^ Since then, a number of small studies have suggested a favorable effect of HVHF in refractory septic shock, mostly in terms of catecholamine requirement,^16^, ^29^, ^30^ but also in terms of oxygenation.^17^, ^31^ The dominant explanation for these effects has been the removal of unselected inflammatory mediators.^19^, ^21^ Alternatively, some suggest that hemodynamic improvement by HVHF depends on the prompt homeostatic restoration of body temperature and extracellular fluid (ECF) composition.^22^ Targeted HVHF could have boosted the benefits of standard HVHF through potential mechanisms given below.

### 4.1 Correction of metabolic acidosis and calcium supplementation

Metabolic acidosis is known to depress myocardial function^32^ and to reduce catecholamine responsiveness in myocardiocytes and vascular myocytes.^33^ Acidemic pulmonary vasoconstriction^32^ may contribute to acute cor pulmonale in acute respiratory distress syndrome.^34^ During circulatory failure a vicious cycle may establish where hypoperfusion leads to metabolic acidosis that further impairs cardiovascular response. However, alkali fluid resuscitation has not consistently translated into positive results likely due to the generation of large amounts of CO_2_, decreased ionized calcium,^33^ and ECF expansion hampering gas exchange and eventually myocardial function itself.^7^ HVHF rapidly restores ECF composition without inducing FO. If insufficient or too slow, bicarbonate can be given externally while UF is added to avoid FO. At the same time minute, ventilation can be transiently increased to clear the extra CO_2_ produced and calcium supplemented to counter the drop in ionic calcium. Such a strategy worked in a rat model of severe lactic acidosis.^35^ This was more purposeful done in our patients during targeted HVHF.

### 4.2 Hyperoncotic albumin fluid resuscitation

One answer to the classical critical care conundrum of fluid resuscitation in the context of leaky capillaries is the use of colloids as resuscitation fluids. Artificial colloids have been abandoned due to side effects, and albumin is the only colloid still in use.^36^ In septic patients a bolus of 200 ml of 20% albumin expands intravascular volume by 430 ml at 30 min.^37^ Besides preload augmentation, albumin has shown positive effects on myocardial contractility in endotoxemic^38^ or cirrhotic myocardial depression^39^ models. The ALBIOS trial showed that in patients with severe sepsis the use of 20% albumin to maintain serum albumin above 3 g/dl was associated with higher mean arterial pressure and less FO; moreover, the septic shock subgroup showed a survival benefit.^40^ In healthy volunteers, 20% albumin produced a larger increase in SI than a fivefold crystalloid bolus while increasing lung diffusion capacity, suggesting balanced salutary hemodynamic and pulmonary effects.^41^ This has led to the concept of 20% albumin “small volume resuscitation.”^42^ The use of large 20% albumin boluses during targeted HVHF may have contributed to the hemodynamic stabilization that averted VA ECMO connection in HVHF‐responders. The fifth patient provides a good example; 400 ml of 20% albumin were given, ITBVI increased, SI increased, HR dropped and lactate washed out (Figure 1 and Table S1).

### 4.3 Fluid overload management

During targeted HVHF not only isovolemic HVHF took place, but large UF amounting to 2.5–5 L was added to give room for bicarbonate, albumin, and immune plasma while sparing FO which has been independently associated with unfavorable outcomes in ICU^43^ and HCPS patients.^14^ As it was seen on patient 5, fluid resuscitation was followed by an exacerbation of lung edema and respiratory failure that could be contained by high PEEP levels (Figure 2 and Table S1). Ultrafiltration allows to dynamically balance cardiac preload optimization and containment of pulmonary flooding as achieved in patients 3 and 4 who slightly increased their ITBVI without increasing EVLWI (Figures 1 and 2, Table S1). Targeted HVHF could provide a mechanism to achieve homeostasis while withholding FO until endothelial tight junctions are restored.

### 4.4 Temperature control

Fever increases oxygen consumption and CO_2_ production.^44^ Convective therapies produce heat loss^22^, ^45^ that could reduce both.^46^ In the context of circulatory failure where oxygen delivery is insufficient to sustain tissue demands, temperature control could help to avoid tissue oxygen debt and restore homeostasis.^44^ In fact, mild hypothermia improved stroke volume, arterial pressure, mixed venous oxygen saturation, and survival in a cardiogenic shock model.^47^ In patients with cardiogenic shock mild hypothermia reduces heart rate and catecholamine requirements while increasing ejection fraction.^48^ Additionally, in patients with septic shock cooling decreased vasopressor requirement and short‐term mortality.^48^ Temperature control could therefore be another potential mechanism of benefit of HVHF. Even though in our series temperature reduction was more prominent in HVHF‐nonresponders, absolute temperature reached was lower in HVHF‐responders (Table S1).

Severe HCPS remains a condition with high lethality. VA ECMO has decreased mortality from 100% to 33% in the most severe cases.^9^ Unfortunately, arterial access, as well as bleeding complications, makes VA ECMO a suboptimal life support technique. In fact, one of our VA ECMO patients had severe vascular morbidity. This highlights the need to explore alternative support measures for severe HCPS. High volume hemofiltration, perhaps with a targeted approach as outlined, could fill this gap. Though provoking, the current report is evidently limited by its retrospective observational nature, describing a clinical practice change along a learning curve based on a small sample of an infrequent disease.

## 5 CONCLUSION

Considering the lack of specific therapy for HCPS, the significant morbidity attributable to VA ECMO, and the ease of HVHF in the ICU setting, our report provides relevant data to consider an early targeted HVHF trial in severe HCPS. Given the uncertainty of the response to HVHF and the mortality of severe HCPS, such a trial should only be performed in centers with VA ECMO capability. The observational nature and small size of the cohort preclude stronger inferences.

## AUTHOR CONTRIBUTIONS

J.G, R.L, M.E, and PA.V. conceptualized the study; J.G, R.L, R.P.‐A, and C.V prepared the methodology; J.G, R.L, R.P‐A, M.E, and PA.V designed the study; R.P‐A, A.S, C.V, A.C, R.L, and J.G managed the data; J.G, R.L, R.P‐A, and M.E formally analyzed the manuscript; J.G, R.L, R.P‐A, A.S, M.E, C.V, A.C, and PA.V. prepared and wrote the original draft; PA.V. acquired the funding for the study.

## CONFLICT OF INTERESTS

The authors declare that there are no conflict of interests.

## ETHICS APPROVAL

The cohort is part of a prospectively obtained database by the Hantavirus program from the Instituto de Ciencias e Innovación en Medicina, Facultad de Medicina Clínica Alemana‐Universidad del Desarrollo. Our local IRB and ethics committee approved this registry (ID19, September 29th 2011).

## Supporting information

Supporting information.

## Data Availability

Data available on request from the authors.
