# Supplementary material for: Targeted high volume hemofiltration could avoid extracorporeal membrane oxygenation in some patients with severe Hantavirus cardiopulmonary syndrome
Source: J Med Virol. 2021 Mar 23;93(8):4738–47. doi: 10.1002/jmv.26930 (PMC8359853; doi:10.1002/jmv.26930)
Supplement: Supplementary file 1 — Supporting information. [file JMV-93-4738-s001.pdf]

Table S1

Changes in laboratory, hemodynamic and transpulmonary thermodilution variables induced by HVHF.

Characteristic	HVHF-nonresponders				HVHF-responders						
	1		2		3			4		5	
**Laboratory variables**	**T-0**	**T-S**	**T-0**	**T-S**	**T-0**	**T-S**	**T-T**	**T-0**	**T-T**	**T-0**	**T-T**
pH	7.31	7.26	7.35	7.38	7.44	7.27	7.59	7.35	7.45	7.13	7.36
Bicarbonate, mmol/L	23	18.5	22.2	23.3	25	17.7	26.4	22.3	26.8	13.7	23.1
Lactate, mmol/L	3.76	7.37	3.44	3.11	2.05	11.7	3.75	3.12	2.46	5.39	1.84
Ionized calcium, mg/dL	4.8	4.8	4.6	5.0	3.9	4.3	4.8	4.6	4.4	4.0	5.2
Hematocrit, %	48	48.9	44.1	47.2	55.8	52.1	39.7	44.3	36.4	47	34
Serum albumin, mg/dL	2.8	2.5	2.9	2.0	3.8	2.7	4.6	2.6	2.3	2.0	3.7
**Vital sings**											
Temperature, °C	39.9	36.9	38.6	36.6	36.7	n/a	n/a	37.4	35.5	37.1	36.3
HR, beats/min	132	140	125	102	98	111	99	96	61	145	113
**Transpulmonary thermodilution variables**											
Stroke index, mL/m^2	18.2	14.3	11.6	9.6	36.4	27.8	50.3	22.5	32.7	29	54.3
ITBVI, mL/m^2	565	451	447	275	742	629	686	467	702	686	1017
SVV, %	18	23	6	10	19	12	9	10	8	7	5
EVLWI, mL/kg	12.4	14.7	20.3	8.8	20.9	18.8	16.5	12	12.8	11.6	13.1

HVHF, high volume hemofiltration; T-0, evaluation prior to HVHF; T-S, evaluation after the standard HVHF run or at the time of connection to VA ECMO; T-T, evaluation after the targeted HVHF run; n/a, non-available; HR, heart rate; ITBVI, intrathoracic blood volume index; SVV, stroke volume variation; EVLWI, extravascular lung water index
